# Insecticide resistant *Anopheles* from Ethiopia but not Burkina Faso show a microbiota composition shift upon insecticide exposure

**DOI:** 10.1186/s13071-024-06638-2

**Published:** 2025-01-20

**Authors:** Netsanet Worku, Antoine Sanou, Juliane Hartke, Marion Morris, Fatoumata Cissé, Salimata Ouédraogo, Madou Tapsoba, Nicola Vallon, Tewodros Debebe Akilu, Ligabaw Worku, Moussa Wamdaogo Guelbeogo, Victoria A. Ingham

**Affiliations:** 1 Institute of Public Health (IPH), College of Medicine and Health Sciences, University of Gondar, PO Box 196, Gondar, Ethiopia; 2 Centre National de Recherche et de Formation sur le Paludisme (CNRFP), Rue 1487 Avenue de la liberté, Ouagadougou, Burkina Faso; 3 Université Yembila-Abdoulaye-Toguyeni (UYAT), 54 Route Bogandé, Fada NGourma, Burkina Faso; 4 University Hospital Heidelberg, Medical Faculty, Centre for Infectious Diseases, Heidelberg University, Im Neuenheimer Feld 324, 69120 Heidelberg, Germany; 5 Vector Biology Department, Liverpool School of Tropical Medicine, Pembroke Place, Liverpool, L35QA UK; 6 BIOMES NGS GmbH, Schwartzkopffstraße 1, 15745 Wildau, Germany; 7 Department of Parasitology, School of Biomedical Sciences, College of Medicine and Health Sciences, University of Gondar, POBox 196, Gondar, Ethiopia; 8 University Joseph KI Zerbo, 03 BP 7021 Ouagadougou, Burkina Faso

**Keywords:** Anopheles, Malaria, Microbiome, Insecticide resistance, Transcriptomics, Microbiota

## Abstract

**Background:**

Malaria remains a key contributor to mortality and morbidity across Africa, with the highest burden in children under 5. Insecticide-based vector control tools, which target the adult *Anopheles* mosquitoes, are the most efficacious tool in disease prevention. Due to the widespread use of these interventions, insecticide resistance to the most used classes of insecticides is now pervasive across Africa. Understanding the underlying mechanisms contributing to this phenotype is necessary to both track the spread of resistance and to design new tools to overcome it.

**Methods:**

Here, we compare the microbiota composition of insecticide-resistant populations of *Anopheles gambiae*, *An. coluzzii* and *An. arabiensis* from Burkina Faso, and in the latter case additionally from Ethiopia, to insecticide-susceptible populations.

**Results:**

We show that the microbiota composition between insecticide-resistant and -susceptible populations does not differ in Burkina Faso. This result is supported by data from laboratory colonies originating in Burkina Faso across two countries. In contrast, *An. arabiensis* from Ethiopia demonstrates clear differences in microbiota composition in those dying from and those surviving insecticide exposure. To further understand resistance in this *An. arabiensis* population, we performed RNAseq and saw differential expression of detoxification genes associated with insecticide resistance and changes in respiration, metabolism and synapse-related ion channels.

**Conclusions:**

Our results indicate that, in addition to changes in the transcriptome, microbiota can contribute to insecticide resistance in certain settings.

**Graphical Abstract:**

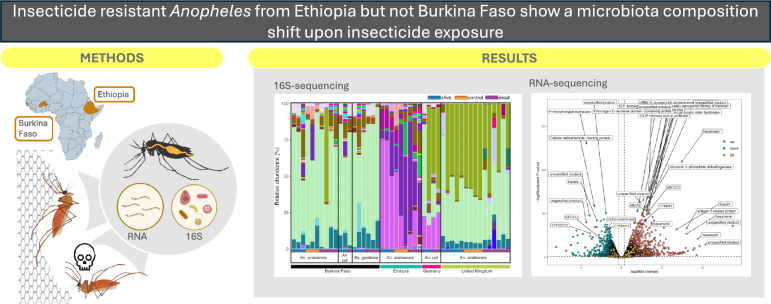

**Supplementary Information:**

The online version contains supplementary material available at 10.1186/s13071-024-06638-2.

## Background

Malaria, caused by the *Plasmodium* parasite and transmitted by *Anopheles* mosquitoes, remains one of the world’s most deadly diseases, with 249 million cases and 609,000 deaths in 2022 [1]. Despite the gains made in malaria control since the implementation of concerted intervention strategies, the downwards trend plateaued from 2015, and since the recent COVID-19 pandemic there has been a clear increase in case numbers [1]. Challenges in reversing this trend and making progress towards elimination have resulted in the World Health Organisation calling for an increase in funding and implementation of new approaches.

Insecticide-based vector control tools, such as indoor residual spraying and insecticide-treated bed nets, are a cornerstone of malaria control programmes; of these, insecticide-treated bed nets showed the most substantial influence on the reduction of case numbers [2]. These interventions directly target the mosquito vector. In Africa, malaria is mainly transmitted by three species within the *Anopheles gambiae* species complex (*An. gambiae*, *An. coluzzii* and *An. arabiensis*) and *An. funestus* [3, 4]. Currently, a limited number of insecticide classes are available for malaria control, the most important of which is the pyrethroid class, which is used on all insecticide-treated bed nets [1]. The selection pressure imposed by pyrethroid insecticides has led to widespread and intense resistance to this chemistry [5]. Indeed, the strength of resistance is such that, in some countries, exposure to pyrethroids has no effect on the longevity of the mosquito vector [6]. In response to escalating resistance, next-generation bed nets have been developed, recommended and deployed, containing pyrethroids and a second chemistry [7, 8]. Furthermore, new ways to administrator insecticides are being explored, such as eave tubes [9] and attractive targeted sugar baits [10], and new chemical classes for indoor residual spraying are now available [11, 12].

Insecticide resistance is a complex phenotype, including increased expression of transcripts involved in metabolic detoxification, single-nucleotide polymorphisms reducing the efficacy of the insecticide, reduced penetration because of thickening of the cuticle and sequestration of the insecticides [13]. Upregulation of metabolic enzymes involves the cytochrome p450 class, which has been shown to directly metabolise several licensed insecticides, including pyrethroids [14–16]. Numerous P450s are upregulated in multiple populations across Africa, including CYP6M2, CYP6P3, CYP9K1 and CYP6AA1 in *An. gambiae* and *An. coluzzii*, CYP6P4 in *An. arabiensis* and CYP6P9a/b in *An. funestus * [14, 17–20]. In addition to cytochrome P450s, GSTs [21], ABC-transporters [22] and most recently UGTs [23] have been implicated in metabolic resistance. Other transcriptomic changes include upregulation of putative insecticide binders, such as the D7 salivary gland proteins [24] and the chemosensory proteins [25]. Outside transcriptomic changes, mutations to the target sites of the insecticide are well characterised. In the *An. gambiae* species complex, mutations in the voltage-gated sodium cannels have been consistently linked with pyrethroid resistance; this includes ‘traditional’ *kdr*, L995F [26] and ‘new’ *kdr* V402L-I1527T [27]. A second known mutation, G119S, in the acetylcholine esterase gene ACE-1 [28] is linked to resistance to both carbamates and organophosphate insecticides.

Although resistance is typically described as a genetic component of the *Anopheles* vector, recent work has shown that the microbiota changes upon insecticide exposure, hinting at the role of these organisms in resistance. Indeed, work on the South and Central American vector, *Anopheles albimanus*, showed significant changes in the cuticle microbiota after pyrethroid exposure and changes in the overall microbiota after organophosphate exposure [29]. In Africa, pyrethroid resistance has been linked to changes in microbiota composition in Cameroon, Kenya and Côte D’Ivoire, whilst laboratory-adapted *An. coluzzii* have been shown to have a changed their microbiota after selection to pyrethroid resistance [30–34]. Furthermore, experimental treatment with antibiotics and spiking with known bacteria show increased pyrethroid tolerance in a laboratory-colonised *An. arabiensis* [35]. Taken together, these data indicate that insecticide resistance may be linked to the mosquito microbiota and that this facet of insecticide resistance could be manipulated for vector control.

In this study, we use 16S sequencing to determine whether the microbiota of laboratory-colonised mosquitoes and field-caught mosquitoes from West and East Africa differ between mosquitoes surviving and dying after exposure to the pyrethroid deltamethrin. In the context of insecticide resistance, comparisons of the microbiota from different regions in Africa, with different species and resistance levels, might provide insight into regional influences on microbiota communities. The laboratory colonies originated from Burkina Faso and were reared at two separate European laboratories (*An. coluzzii,* Germany, and *An. arabiensis*, UK), mosquitoes from Burkina Faso represent all three species of the *An. gambiae* species complex, and those from Ethiopia were *An. arabiensis*. Here, we show that *An. arabiensis* from Ethiopia have distinct microbiota profiles for surviving and dying mosquitoes, whilst those from Burkina Faso and the two laboratories do not. To further understand insecticide resistance in this *An. arabiensis* population, we performed RNAseq and saw upregulation of insecticide resistance-related genes and overall changes in genes related to respiration. Integration of these data with a second population from Ethiopia highlights key detoxification genes in this region. Further comparisons with *An. arabiensis* from Burkina Faso show a large difference in the transcriptomic profile but nevertheless highlight four core detoxification genes overexpressed across Africa.

## Methods

### Field collections

Mosquito collections were carried out in both Ethiopia and Burkina Faso respectively using larval sampling. Mosquito larvae were collected from 7 July to 2 August 2021 in Tiefora in Burkina Faso (latitude 10.62411667, longitude − 4.55335) characterised by high use of insecticides in agriculture, mainly lambda-cyhalothrin (a pyrethroid) and pyrethroid-based bed nets (deltamethrin, alpha-cypermethrin and permethrin). Since 2019, pyrethroid plus PBO nets have been deployed in this region. From 28 July to 6 August, field sampling was conducted in Bahir Dar (latitude 11.588790, longitude 37.3888119) in Ethiopia, where using deltamethrin (pyrethroid) in LLIN was common, as well as using pirimiphos-methyl (organophosphate) and bendiocarb (carbamate) in IRS [36].

### Mosquito rearing

Mosquitoes were reared under standard insectary conditions of 28°C ± 2  C with a 12:12 h light:dark cycle with 1 h dawn:dusk. Larvae were fed ground fish food and upon emergence transferred to cages and maintained on 10% sucrose solution. Banfora (*An. coluzzii*) and Gaoua (*An. arabiensis*) were reared in insectaries at Heidelberg University Hospital (UKHD) and Liverpool School of Tropical Medicine (LSTM), respectively; each strain was originally colonised from Burkina Faso in 2015 and 2018, respectively [37, 38]. Moz (*An. arabiensis*) was originally colonised in 2009 from Mozambique [39] and reared at UKHD. The field-caught mosquitoes were reared in insectaries at University of Gondar (*An. arabiensis*) and Centre National de Recherche et de Formation sur le Paludisme (CNRFP) (*An. gambiae, An. coluzzii* and *An. arabiensis*).

### Insecticide susceptibility tests using adult mosquitoes

Mosquitoes were reared and, at 3–5 days old, presumed mated, were exposed to 1 ×, 5 × and 10 × diagnostic doses of 0.05% deltamethrin for 1 h using standard WHO tube tests [40] to determine the dose that gave ~ 70% mortality at 24 h. After exposure to the appropriate dose (5XDD in field samples, 1XDD at LSTM and UKHD), mortality was recorded 24 h later. Control mosquitoes were from the same cohort, unexposed and alive at the time of processing. Mosquitoes were then individually stored at – 20  C and their live and dead phenotype recorded. Mosquitoes from University of Gondar were shipped to CNRFP for further processing to ensure consistency of field samples. Extractions were done separately at CNRFP, UKHD and LSTM following the below protocol.

### Sample processing

Mosquitoes were individually thawed and 250 µl of 70% alcohol added followed by vortexing for 10 s to remove the surface microbiota; 250 µl of sterile distilled water was then added, and the samples were vortexed for a further 10 s. The samples were then gently rinsed and allowed to dry. Once dry, the head and thorax were separated from the abdomen, and each was stored in separate tubes for further processing.

### Species identification and molecular analysis

DNA was extracted from the heads and thoraces by boiling in STE buffer for 15 min at 95°C. The subsequent DNA from individual mosquitoes underwent species identification through SINE200 PCR following a previously published protocol [41]. To determine the frequency of *kdr*-L995F [42] and ACE-1 G119S [43], additional PCRs were performed following standard protocols.

### DNA extraction for 16S sequencing

Mosquito abdomens were then pooled into groups of five by species, alive:dead phenotype and location. DNA was then extracted from individual mosquitoes using the LIVAK protocol as previously published [44]. Briefly, LIVAK buffer pH 7.5 (1.6 ml 5M NaCl, 1.7 ml 0.5 M EDTA, 2.4 ml 1 M Tris, pH 7.5, 5 ml 10% SDS and up to 100 ml ddH_2_O) was heated to 65°C. Mosquitoes were then homogenised in 100 µl LIVAK buffer and heated for 30 min at 65°C; 14 µl of 8 M potassium acetate was added, mixed and left on ice for 30 min. After centrifugation, the supernatant was collected and 200 µl of 99.9% ethanol added. DNA was then precipitated and resuspended in 50 µl of ddH_2_O. DNA was then shipped to BIOMES GmbH for 16S sequencing; sample information can be found in Supplementary Table 6.

### 16S analysis

16S abundance tables were provided by BIOMES GmbH and analysed using the vegan package [45] in R following previously published methodology [33]. Briefly, alpha diversity was analysed using an ANOVA on the Shannon index followed by Tukey multiple comparisons for country, species and alive/dead status. Beta diversity was calculated using Brays-Curtis to account for OTU abundance, following by a permuted ANOVA (*n *= 1000) using country and alive/dead groupings. Beta dispersion was explored using a permutation test (*n* = 1000) on nMDS for country pools.

### RNA extraction

Total RNA was extracted from the field mosquitoes from Bahir Dar, Ethiopia (ETH), after exposing them as described above, as well as from susceptible *An. arabiensis* maintained at UKHD (Moz) [37]. RNA was extracted from five pooled adults using the PicoPure RNA extraction kit following the manufacturer’s instructions. Total RNA quantity and quality were checked on a NanoDrop and Bioanalyzer, respectively, before being submitted to Eurofins Genomics for polyA enrichment-based RNA sequencing.

### RNAseq analysis

The fastq files were aligned to *An. arabiensis* DONGALA assembly using Hisat2 [46] with default parameters. featureCounts [47] was then used to extract read counts for each gene. Count files were analysed using the DESeq2 package [48] in R as previously described [33]. GO and KEGG enrichments were performed within VectorBase [49] with *p*-values taken with Bonferroni correction. Fastq files for RNAseq of *An. arabiensis* from Burkina Faso and from Ethiopia (PERM vs DON) were retrieved from SRA (PRJNA780362, PRJNA730212). Families were extracted from VectorBase using PFAM IDs as follows: PF00067, PF00201, PF00135, PF00005, PF12848, PF03392, PF00011, PF03722, PF00372 and PF01395.

To confirm frequencies of SNPs in our samples from Ethiopia, variants were called using bcftools v1.21 and allele frequencies at the positions of *kdr*-L995F, *kdr*-L995S, ACE-1 G119S and GSTE2 L119V were checked.

## Results

### Species identification of field samples

Species identification was performed on a total of 441 individual mosquitoes collected in Tiefora, Burkina Faso and Bahir Dar, Ethiopia, consisting of 248 and 107 samples, respectively. The Burkina Faso collections consisted of 134 (54%) *An. arabiensis*, 21 (8.5%) *An. coluzzii*, 92 (38.1%) *An. gambiae* and 1 (0.4%) *An. gambiae* x *An. coluzzii* hybrid. In contrast, the Bahir Dar samples were all *An. arabiensis* (107 samples). Table 1 shows the number of control unexposed mosquitoes and those alive and dead following exposure to the pyrethroid deltamethrin.

**Table 1:** Breakdown of the mosquito samples by species, mortality status and country

Country/species	Control		Control total	Exposed			Exposed total	Total
	Alive	Dead		Alive	Dead	24-h Mortality (%)		
**Burkina Faso**	**58**		**58**	**88**	**102**	**54**	**190**	**248**
*Anopheles arabiensis*	48		48	18	68	79	86	134
*An. coluzzii*	4		4	12	5	29	17	21
*An. gambiae*	6		6	57	29	34	86	92
Hybrid (*An. gambiae* x *An. coluzzii)*				1		0	1	1
**Ethiopia**	**32**	**2**	**34**	**15**	**58**	**79**	**73**	**107**
*An. arabiensis*	32	2	34	15	58	79	73	107
Grand total	**90**	**2**	**92**	**103**	**160**	**61**	**263**	**355**

Table 1 shows the country and species for control unexposed mosquitoes, alive, dead and total and those that were exposed to 5XDD deltamethrin through WHO tube bioassay, alive, dead and total. The final column shows the total number of mosquitoes tested for each row. The final row shows the total across both countries. Bold values show total numbers per country and across countries

### Resistance levels

**Fig. 1:**
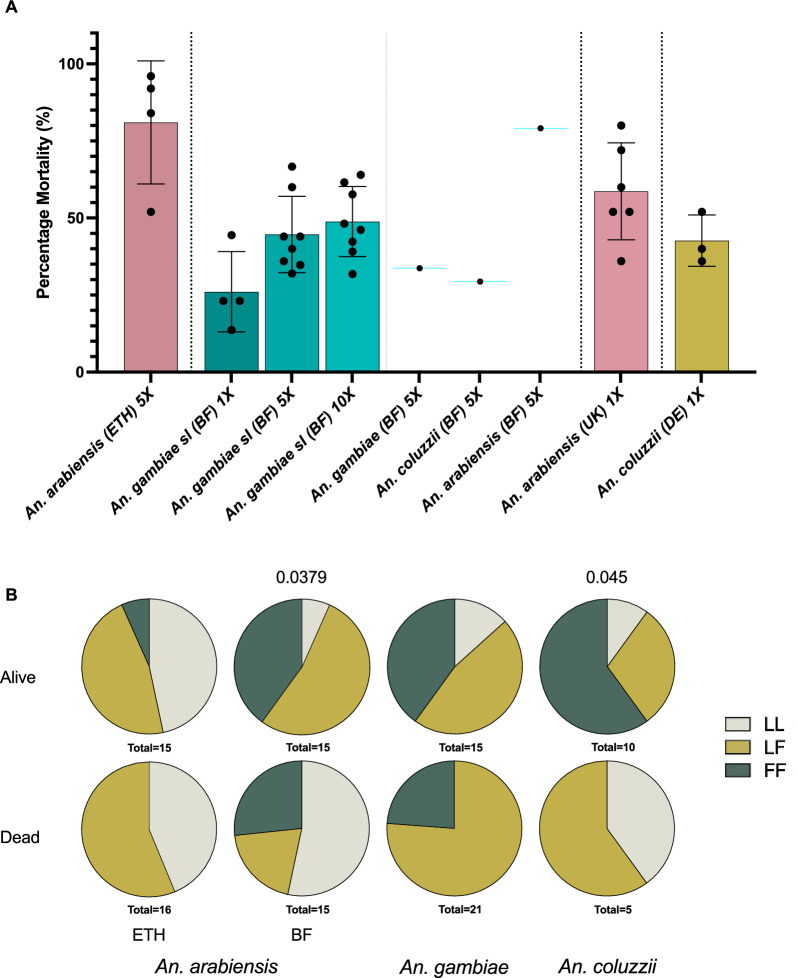
Insecticide resistance status. **A** Mortality data for each population used in the 16S experiments to 0.05% (1X), 0.25% (5X) or 0.5% (10X) deltamethrin in WHO tube assays for each country. As the samples from Burkina Faso underwent species identification after testing, they are displayed as a single line. In all other cases, points represent 25 mosquitoes tested together in one WHO tube. Error bars show standard deviation. **B** *kdr*-L995F frequency in the field populations used for 16S. Total number of individuals given under each pie chart. *ETH* Ethiopia, *BF* Burkina Faso, *DE* Germany. Significant *p* values are indicated above the pie charts comparing alive:dead allelic frequency

Resistance to deltamethrin (Fig. 1A) was highest in field-caught *An. gambiae* with 66.3% of the mosquitoes surviving 5XDD exposure, in contrast to 20.9% survivorship of *An. arabiensis. Anopheles coluzzii* from Burkina Faso also showed similarly high survivorship (70.6%) but with only 17 samples. *Anopheles arabiensis* samples from Ethiopia showed similar resistance levels to *An. arabiensis* in Burkina Faso with 20.5% surviving 5XDD. The laboratory colonies were exposed to 1XDD of deltamethrin, with 41% *An. arabiensis* surviving and 67.3% of *An. coluzzii*. Each field-caught individual used for subsequent microbiota work was then assessed for the presence of *kdr*-L995F and ACE-1 G119S mutations (Fig. 1B); *kdr-*L995F was present in 42.6% of the *An. arabiensis* from Ethiopia (29.4% in dead and 33.3% in alive) and ACE-1 at 6.4**%** (3% in dead and 10% alive). In Burkina Faso, *kdr*-L995F was present at 54.4% in *An. arabiensis* (66.7% alive and 36.7% dead) and was significantly associated with survival (*p*_χ2_ = 0.0379); *An. coluzzii* at 60% (75% alive and 30% dead, *p*_χ2_ = 0.045) and *An. gambiae* at 62.5% frequency (63.3% alive and 61.9% dead, *p*_χ2_ = 0.88). L995S was not tested here but is likely present in *An. arabiensis* from Burkina Faso [37]. ACE-1 varied at lower frequency compared to *kdr*-L995F across all Burkina samples, with 4.4% in *An. arabiensis*, 3.3% in *An. coluzzii* and 19.5% in *An. gambiae*.

### Microbiota diversity

**Fig. 2:**
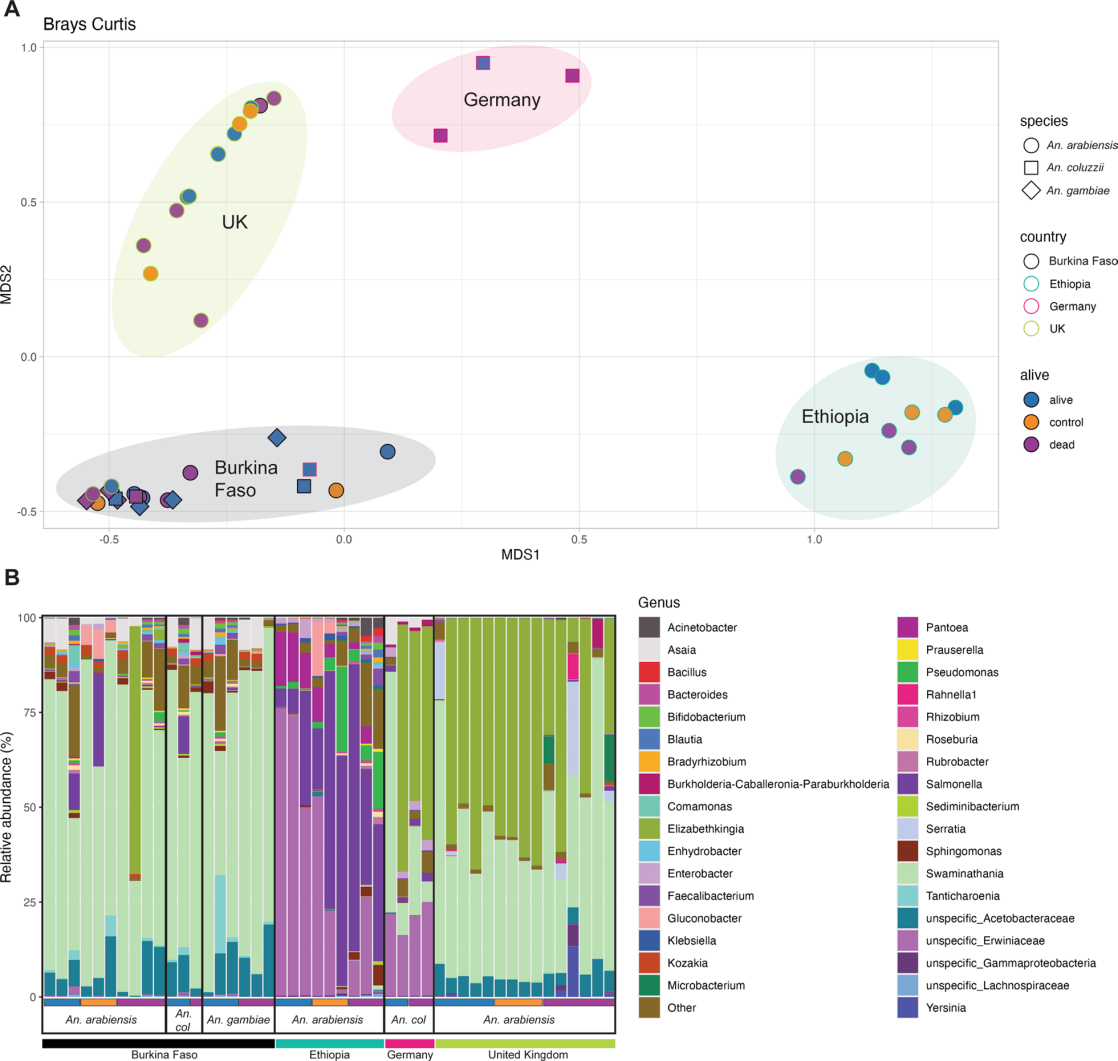
16S microbiota diversity. **A** Brays-Curtis multidimensional scaling plot showing MDS1 (x-axis) and MDS2 (y-axis) for the calculated abundances. Each point represents one pool of five individuals; the colour represents alive (green) and dead (pink) after deltamethrin exposure, and unexposed controls (yellow). The shape indicates species *Anopheles arabiensis* (circle), *An. gambiae* (diamond) and *An. coluzzii* (square). The shape outline denotes the country: Burkina Faso (black), Ethiopia (turquoise), Germany (pink) and the UK (green). Ellipses represent each country cluster and are labelled as such. **B** Relative abundance (% y axis) plots for each pool of five individuals (x axis) at genus level. Label acronyms are as follows: col = *An. coluzzii*. Alive samples are denoted by a blue bar, control a yellow bar and dead a pink bar immediately below the x-axis. Countries are denoted by a bar and coloured as in A

16S sequencing was then performed on live and dead mosquitoes from pools of each species from each location and relative abundance calculated. No differences in alpha diversity were observed, indicating no difference in operational taxonomic unit (OTU) richness; however, beta diversity was significantly different between the countries and the interaction term of countries and alive/dead status (P_ANOVA_ = 0.001 and 0.008 respectively) signifying diversity between these factors. No differences were observed for beta dispersion across the countries, demonstrating similar variances between groups. A Bray-Curtis multidimensional scaling plot (Fig. 2A) shows that the samples largely separate on location, with some notable exceptions. Several samples from the *An. arabiensis* colony and one sample from the *An. coluzzii* colony overlap with the Burkina Faso samples, whilst one *An. arabiensis* pool from Burkina Faso overlaps with those from the *An. arabiensis* colony. This may indicate some conservation of original microbiota stochastically in individuals across many generations and across multiple localities. There is no clear distinction between the samples from Burkina Faso based on species; this lack of separation might be expected, as the individuals, despite originating from different larval environments, were later pooled. Indeed, sharing a niche during the aquatic stage has been shown to significantly influence microbiota composition [50]. Interestingly, whilst the samples from Burkina Faso and the colonies show no separation of those mosquitoes surviving or dying after insecticide exposure, there is clear separation of the Ethiopian samples, indicating that microbiota composition is linked to insecticide resistance in these *An. arabiensis* samples. These samples were collected from the same collection site, potentially explaining the clearer association.

The abundance plots (Fig. 2B) show 2–6 highly abundant genera across each population. *Anopheles arabiensis* from Ethiopia are dominated by *Erwiniaceae*, which align to an unspecified taxon, *Salmonella* and *Pantoea*. Six OTUs have > 10% abundance in the colonised *An. coluzzii*, including *Asaia*, *Salmonella*, an unspecified *Erwiniaceae*, *Swaminathania*, *Elizabethkingia anophelis* and *Enterobacter amnigena* whilst *An. coluzzii* from Burkina Faso are dominated by *Swaminathania*, with *Asaia* and *Salmonella* having > 5% abundance. Similarly, *An. gambiae* from Burkina have these three OTUs with > 5% abundance but uniquely have *Tanticharoenia*. *Anopheles arabiensis* from Burkina Faso again shows a similar dominant taxon, overlapping entirely with the other species. The *An. arabiensis* colony is dominated by two taxa: *Elizabethkingia anophelis* and an unspecified *Swaminathania*.

### Bacteria associated with survival to pyrethroid exposure

**Fig. 3:**
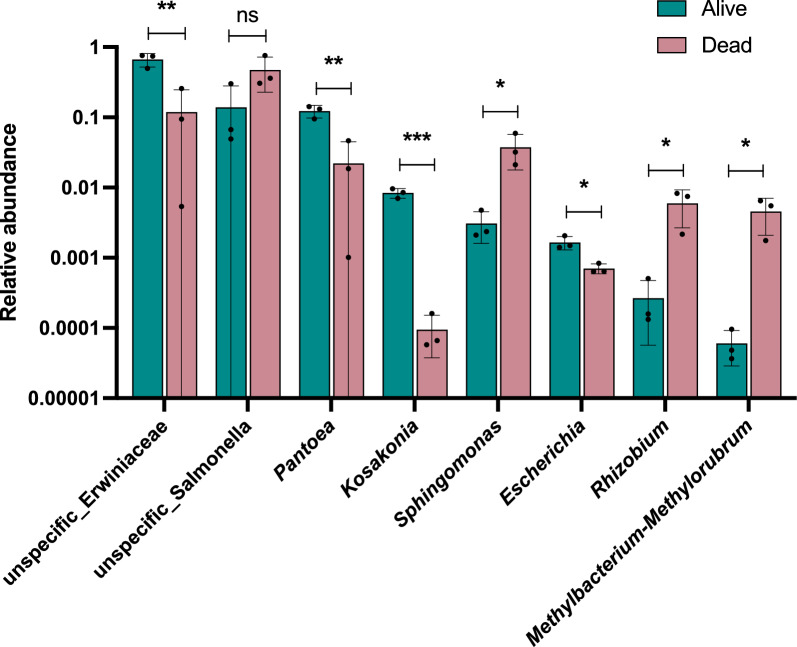
Bacterial OTUs associated with pyrethroid resistance in *Anopheles arabiensis* from Ethiopia. Relative abundance (y axis) of genera (x-axis), alive (green) and dead (pink). Statistical significance calculated by ANOVA, *p < 0.05, **p < 0.01, ***p < 0.001. Error bars represent standard deviation

To explore any association with insecticide resistance, the pools were split by country and association with survival assessed for OTUs representing > 1% of the overall abundance. Field-caught samples from Burkina Faso had one genus associated with survival, *Sphingomonas*, which was significantly more abundant in survivors (p_ANOVA_ = 0.029). Similarly, the *An. arabiensis* colony had a low-abundant unspecified *Gammaproteobacteria*, which was more abundant in dead mosquitoes (p_ANOVA_ = 0.03). Unlike these tenuous associations, the field-caught *An. arabiensis* from Ethiopia had clear associations with bacterial genera showing significant association with survivorship. As for Burkina Faso, *Sphingomonas* was significantly associated with exposure (p_ANOVA_ = 0.045); however, it was found at higher abundance in dead mosquitoes. Perhaps the most interesting is a highly significant association of *Pantoea* with survival (p_ANOVA_ = 0.0067) at > 12% abundance in alive mosquitoes compared to ~ 2% in dead mosquitoes. Similarly, the unspecified *Erwiniaceae* is at ~ 66% abundance in live mosquitoes and 12% in dead mosquitoes (p_ANOVA_ = 0.0082), whilst *Salmonella* is at ~ 14% in alive mosquitoes and 47% in dead mosquitoes, although this is not significant because of high variation (p_ANOVA_ = 0.11) (Fig. 3). The other genera with significantly higher abundance in survivors include *Escherichia* and *Kosakonia*, whilst *Rhizobium* and *Methylbacterium-Methylorubrum* are more abundant in dead mosquitoes; however, the abundance of all of these genera is on average < 1%, so their relationship to IR at such low levels is unclear. In addition to the resistance phenotype itself, we cannot rule out that the abundance differences might be related to physiological processes after death or to direct effects of the insecticide exposure. However, the grouping of control mosquitoes with alive/dead in Ethiopia indicates that limited change occurred in the microbiota after death.

### Characterisation of insecticide resistance in *An. arabiensis* from Ethiopia

**Fig. 4:**
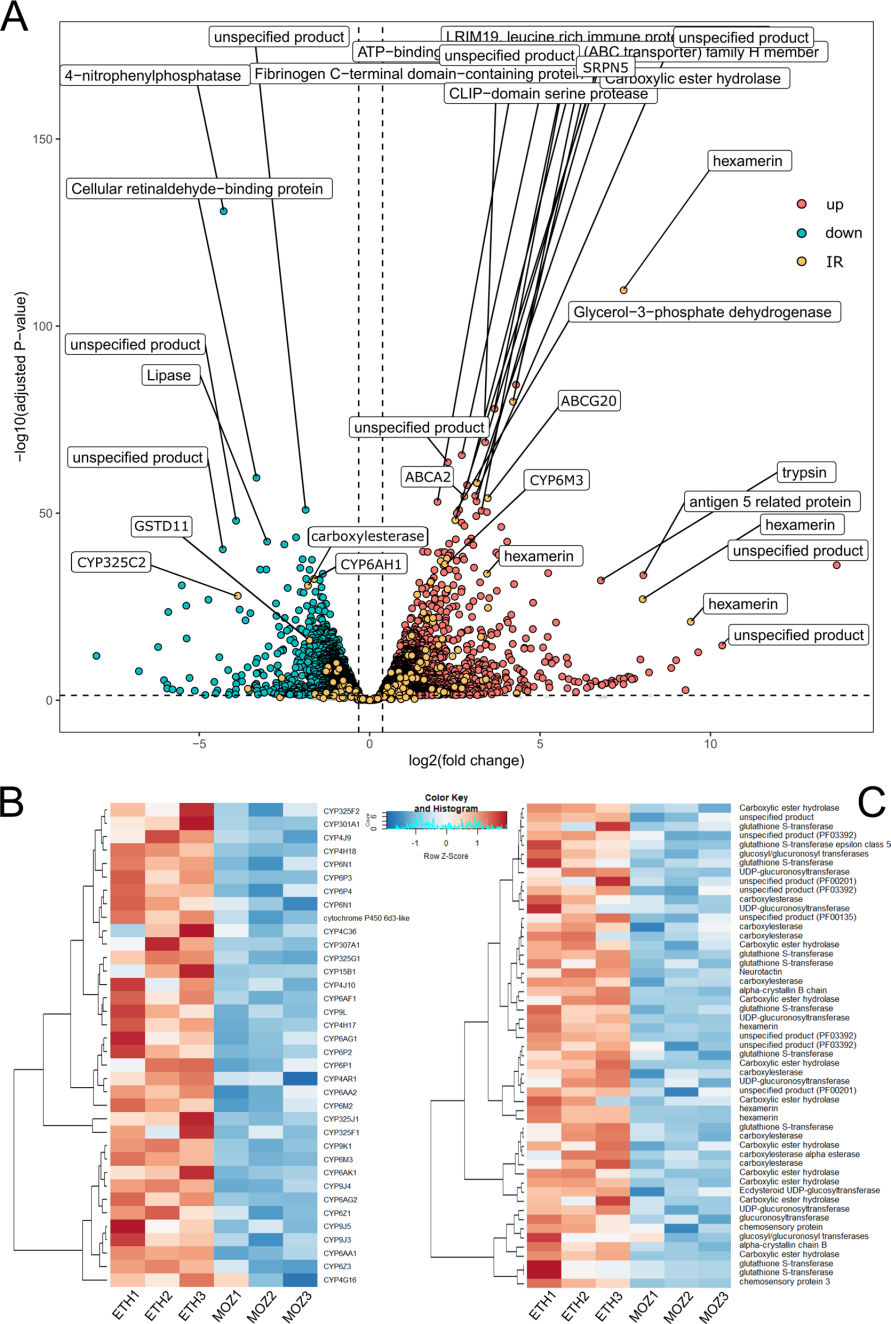
Gene expression changes in *Anopheles arabiensis* from Bahir Dar. **A** Volcano plot showing significantly up- (pink) and downregulated (blue) genes, with genes from members of the ABC transporter, cytochrome P450, chemosensory, carboxylesterase, glutathione-S-transferase and uridine diphosphate-glycosyltransferase families highlighted in orange. **B** Normalised count data for the Bahir Dar (ETH) samples compared to the susceptible Moz for all significant cytochrome p450s. **C** Normalised count data for the Bahir Dar (ETH) samples compared to the susceptible Moz for significant genes from all other resistance-related families. Red indicates higher read count, and blue lower, with a normalised Z score of − 1 to 1

To determine the mechanisms of genetically driven pyrethroid resistance in the field-caught *An. arabiensis* from Ethiopia, RNAseq was performed. PCA demonstrated separation of field mosquitoes from Bahir Dar and the laboratory-susceptible Moz (Supplementary Fig. 1). In total, 2666 genes (26.3%) were significantly overexpressed and 2958 (29.1%) were significantly downregulated (Fig. 4A, Supplementary Table 1). GO terms show significant enrichment in numerous areas, including cellular respiration (*p* = 6.07e−7), ATP synthase (*p* = 6.15e−3), oxidoreduction activity (*p* = 2.09e−5), ion transport (*p* = 1.31e−9) and synapse (*p* = 1.21e−3). Four MetaCyc pathways showed significant enrichment, including lipoprotein post-translational modification (*p* = 1.25e−5), aerobic respiration (*p* = 4.07e-3), Fe(II) oxidation (*p* = 6e−3) and plasmalogen biosynthesis (*p* = 1.47e−2) (Supplementary Table 2). Of the detoxification genes, 23 ABC transporters, 33 cytochrome P450s, 10 GSTs, 5 COEs and 2 UGTs are overexpressed (Figs. 4B-C). These genes include CYP9K1, CYP6P4, CYP6AA1, CYP6Z3 and CYP6M2, which are all known pyrethroid metabolisers in the *An. gambiae* complex [16, 18, 19, 51], and CYP4G16, linked to cuticular thickening [52]. Additionally, in the syntenic ortholog of ABCH2 [53], 3 CSPs [25], 3 alpha-crystallins, 3 hexamerins [14] and 24 cuticular genes are overexpressed (Supplementary Fig. 2). AARA016988 is the homologue of the hexamerin AGAP001345 [14] and is the second most highly significantly over-expressed gene at 175x, whilst AARA016998, the homologue of SAP2 [25], is the fourth with a fold change of 18.45. ABCA7 and ABCG2 homologues are present in the top 20 significantly overexpressed genes, whilst 5 of the top 20 are serine proteases. Variant calling resulted in the confirmation of *kdr*-L995F in the Ethiopia samples in heterozygous form. The other analysed mutations were not found to be present.

GO term enrichments of significantly downregulated genes include nucleic acid binding (*p* = 2.19e−17), gene expression (*p* = 8.55e−31), cellular response to stress (*p* = 5.17e−13) and ribosome biogenesis (*p* = 8.07e−4). The KEGG pathway related to biotin metabolism (*p* = 1.88e−3) is also significantly enriched (Supplementary Table 2). Taken together, these indicate large metabolic changes between these populations.

### Comparison of *An. arabiensis* from Ethiopia and Burkina Faso

A prior dataset of permethrin-resistant *An. arabiensis* from Asendabo, approximately 425 km further south [54], was compared with the results from Bahir Dar. Across both sites, 1443 genes were significant, 607 consistently upregulated and 412 consistently downregulated (Supplementary Table 3). Consistently upregulated genes include CYP6P4, CYP6M2, CYP6P3, CYP9J5 and CYP9K1, all previously implicated in pyrethroid resistance [16, 51, 55, 56]. Furthermore, GSTE2, GSTE7, GSTD3, a chemosensory protein homologue and two ABCG transcripts are included in this list. Enrichments include oxidoreduction-driven active transmembrane transporter activity (*p* = 2.09e−17), oxidoreductase activity (*p* = 1.61e−12), electron transfer activity (*p* = 3.82e−16), cellular respiration (*p* = 1.82–20) and mitochondria (*p* = 2.89e−11). Consistently downregulated genes include response to stress (*p* = 1.61e−2).

Similarly, previously published data are available for *An. arabiensis* from Gaoua, located in southwest Burkina Faso [57]; this was compared to the Ethiopian samples (Supplementary Table 4). A pairwise comparison with the samples from Bahir Dar shows 2762 genes are significant across both sites; of these, 209 are commonly upregulated and 176 are commonly downregulated. Consistently upregulated genes include CYP6AA2, CYP6AG2, CYP6AK1, CYP6P1 and CYP6P4. The other detoxification family members include ABCA2, GSTE7, GSTS1, GSTMS3 and a UGT (AARA006222). Enriched GO terms relate to transporter activity (*p *= 4.95e−2).

A three-way comparison shows 726 genes commonly differentially expressed, of which 55 are commonly upregulated and 22 commonly downregulated (Supplementary Table 5). GSTE7, CYP6AK1, CYP6P4, a UGT (AARA006222), one COE (AARA016325) and multiple serine protease-related proteins are commonly overexpressed. The consistent overexpression in these vastly different populations indicates a key role of insecticide resistance in these populations.

## Discussion

Resistance to pyrethroid insecticides is a complex phenotype, and whilst metabolic and target site changes are relatively well understood, changes in the microbiota are just now beginning to be explored. In this study, we show that microbiota changes may be more important in specific populations; we further characterise resistance in *An. arabiensis* from Bahir Dar and show changes in known resistance-associated transcripts as well as large changes in respiration-related genes, which are also evident in a prior RNAseq study on an *An. arabiensis* population from Ethiopia [43]. Taken together, these results indicate that these mosquitoes potentially rely on a mix of genetic and non-genetic factors of insecticide resistance, putatively because of a commensal association with local bacteria which can complement degradation of insecticides in populations with lower levels of resistance.

Bioassay results here show that Ethiopian *An. arabiensis* have similar levels of pyrethroid resistance to the Burkinabe counterparts but significantly lower than those of *An. gambiae* or *An. coluzzii*. These findings are in line with published literature showing that *An. arabiensis* generally has lower levels of resistance [58, 59], likely due to a lower propensity for anthrophilic behaviour. *Kdr*-West (L995F) was present at high frequency in both countries, with higher levels in Burkina Faso; however, *kdr*-East (L995S) was not assessed here and so correspondingly high levels of this genotype in Bahir Dar cannot be ruled out. Data collected in 2023 indicate the frequency of L995S is 5% in *An. arabiensis* collected from the Tiefora site, Burkina Faso (Sanou, unpublished). Colony mosquitoes from both the UK and Germany are maintained under regular selection pressure [38], and thus high levels of resistance are expected. Interestingly, *An. arabiensis* from Burkina Faso showed a significant association of L995F and survivorship, which was lacking in the samples from Ethiopia, in line with previous data [60]. We postulate that the differences seen in dependency on *kdr* between the countries despite similar levels of mortality might be due to a great reliance on metabolic resistance in the Ethiopian populations, which could lead to a breakdown of the insecticide before reaching the target site. These assumptions are based on two aspects of pyrethroid resistance: the apparent spread of I1527T-V402L in *An. coluzzii* despite this mutation conferring less protection against pyrethroid insecticides [61, 62] and the upregulation of 33 cytochrome P450s in the population from Bahir Dar compared to 13 from the population in Burkina Faso [37].

The microbiota composition observed in the populations of this study aligns well with previous publications, featuring symbionts commonly found in *Anopheles* mosquitoes such as *Pantoea*, *Elizabethkingia anophelis, Asaia* and *Serratia* [30–34, 63]. Several bacterial genera are found across all populations and notably different species here, likely because of previous reports of selectivity in bacterial colonisation of the adult gut and natural occurrence of soil bacteria [50, 63]. Furthermore, previous reports have demonstrated that mosquitoes from similar habitats share a portion of their microbiota [63, 64]. The populations described in this study are dominated by one or two OTUs, with many having < 1% abundance, in agreement with a deep-sequencing study across multiple mosquito species in Kenya [64]. Surprisingly, several pools of mosquitoes from the UK and Germany overlap with Burkina Faso samples, indicative of a stochastic maintenance of field-like microbiota. This requires further investigation as it contradicts the expectation that microbiota are largely determined by larval environment [65], although vertical transfer of bacteria within a population has been suggested previously [66].

Here, we show that *An. arabiensis* from Ethiopia have a significant association of pyrethroid survivorship with microbiota composition that was absent in their Burkinabe counterparts and in the other analysed species from Burkina Faso. *Erwiniaceae, Pantoea, Kosakonia* and *Escherichia* are more abundant in mosquitoes surviving pyrethroid exposure. These OTUs are thus potentially involved in conferring some level of pyrethroid resistance in *An. arabiensis* from Ethiopia. Unlike previous studies, we found no association of *Serratia* or *Asaia* with pyrethroid resistance in this population [32, 34], potentially because of species or locality differences. *Pantoea* is a known insecticide-degrading bacteria [67] and was shown to be at significantly higher levels in those mosquitoes surviving exposure, as seen previously [29]. Similarly *Escherichia* has previously been linked to fenitrothion [30] and deltamethrin [32] resistance and has been shown to naturally metabolise carbamate insecticides [68]. *Kosakonia* has not previously been linked to insecticide resistance in pests but is associated with rice paddy fields and organophosphate remediation [69] and has been shown to inhibit trypanosome infection in tsetse flies [70]. Gut microbiota have been shown to affect insecticide resistance also indirectly by altering the expression of detoxification genes in *Aedes* mosquitoes [66], although this was linked to *Serratia*, which we have not found to be implicated in the resistance status here. It thus seems more likely that any putative connection between bacterial genera and resistance status in this study is conferred by direct metabolism of the insecticide.

Mosquitoes from Bahir Dar have previously been shown to be resistant to pyrethroid insecticides [60] but the underlying mechanisms remained unstudied. Here, we show that the mechanisms of insecticide resistance are consistent with other study sites in Ethiopia and broadly across Africa. For example, the most highly overexpressed genes include a hexamerin [14] and a SAP2 homolog [25], indicating that these families may be important in *An. arabiensis* populations in addition to *An. coluzzii*. Furthermore, overexpression of key pyrethroid-metabolising cytochrome p450s such as CYP6P4 [19], CYP9K1 [51] and CYP6P3 [55] have previously been demonstrated in resistant *An. arabiensis* species. Interestingly, ABCH2, which has been shown to reduce uptake of pyrethroid insecticides in *An. coluzzii* [53], is also overexpressed in this population. The importance of the overexpression of these candidates is underlined by integration of RNAseq data available for *An. arabiensis* from a second site in Ethiopia [54], where similar patterns of gene expression are seen. This second site, Asendabo, is characterized by the use of bendiocarb by the National Malaria Control Programme. Furthermore, organophosphate and pyrethroid resistances have been reported in *An. arabiensis* from this region [60]. In contrast, very few genes are commonly overexpressed in the Burkina Faso *An. arabiensis* [57], suggesting local adaptation to insecticide pressure. Within the Gaoua region, pyrethroids are used in agriculture as well as on bed nets. Distribution of pyrethroid plus PBO nets only started in 2022. Just 55 genes are commonly overexpressed in these populations, which may be unsurprising given the extreme geographical distance and thus likely lack of inbreeding. Nevertheless, key detoxification genes such as CYP6P4, CYP6AK1, GSTE7 and a carboxylesterase may display convergent evolution of overexpression, indicating a key role in pyrethroid resistance in *An. arabiensis*. Temporal separation of these samples (2021 here, 2017 in the second Ethiopia study and 2018 in Burkina Faso) may confound these data as insecticide resistance is likely a continuously evolving trait.

Recent work has linked increased respiration with insecticide resistance [33], consistent with enriched ontology terms both within the Bahir Dar RNAseq produced here and in the integrated Ethiopian data; this again suggests that resistance results in a higher respiratory rate either causally or as a result of this phenotype. If these changes result in differences in underlying reactive oxygen and nitrogen species, as previously shown [71], this could impact both vector competence and microbial colonisation through differential bacterial resistance to ROS killing in long-term commensal bacteria [72, 73].

## Conclusions

The data presented here show that the microbiota may play a role in insecticide resistance in certain settings. We further demonstrate that *An. arabiensis* from within Ethiopia show similar transcriptomic changes resulting in insecticide resistance; however, the number of genes consistent with *An. arabiensis* from Burkina Faso are few. Several caveats remain regarding the conclusions drawn here and other studies. First, a causal relationship between pyrethroid survival and the microbiota must be shown utilising metabolism studies or microbiota transplant. Furthermore, validation of key candidates across multiple populations from vastly different geographies should be demonstrated. Finally, integrating transcriptomic data with microbiota data by directed studies after transplant would give additional insights into whether the microbiota directly impact the mosquito transcriptome in relation to pyrethroid resistance. Nevertheless, taken together, our data demonstrate both site-specificity and cross-country commonalities in resistance, underlining the necessity to test new insecticide products across multiple localities.

## Supplementary Information

Additional file 1. Supplementary Figures

Additional file 2.

Additional file 3.

Additional file 4.

Additional file 5.

Additional file 6.

Additional file 7.

## Data Availability

Data are provided within the manuscript or supplementary information files.

## References

[1] WHO Organization. World Malaria Report. Geneva: WHO; 2023.

[2] Bhatt S, Weiss DJ, Cameron E, Bisanzio D, Mappin B, Dalrymple U, et al. The effect of malaria control on *Plasmodium falciparum* in Africa between 2000 and 2015. Nature. 2015;526:207–11. 26375008 10.1038/nature15535 PMC4820050

[3] Sinka ME, Bangs MJ, Manguin S, Coetzee M, Mbogo CM, Hemingway J, et al. The dominant *Anopheles* vectors of human malaria in Africa, Europe and the Middle East: occurrence data, distribution maps and bionomic précis. Parasites & Vectors. 2010. 10.1186/1756-3305-3-117. 10.1186/1756-3305-3-117 PMC3016360 21129198

[4] Coetzee M, Hunt RH, Wilkerson R, Della Torre A, Coulibaly MB, Besansky NJ. *Anopheles coluzzii* and *Anopheles amharicus*, new members of the *Anopheles gambiae* complex. Zootaxa. 2013;3619:246–74. 26131476

[5] Ranson H, Lissenden N. Insecticide resistance in African *Anopheles* Mosquitoes: a worsening situation that needs urgent action to maintain malaria control. Trends Parasitol. 2016;32:187–96. 26826784 10.1016/j.pt.2015.11.010

[6] Hughes A, Lissenden N, Viana M, Toe KH, Ranson H. *Anopheles **gambiae* populations from Burkina Faso show minimal delayed mortality after exposure to insecticide-treated nets. Parasites Vector. 2020. 10.1186/s13071-019-3872-2. 10.1186/s13071-019-3872-2 PMC6954553 31924276

[7] Messenger LA, Matowo NS, Cross CL, Jumanne M, Portwood NM, Martin J, et al. Effects of next-generation, dual-active-ingredient, long-lasting insecticidal net deployment on insecticide resistance in malaria vectors in Tanzania: an analysis of a 3-year, cluster-randomised controlled trial. Lancet Planetary Health. 2023;7:e673–83. 37558348 10.1016/S2542-5196(23)00137-7

[8] Hien SA, Soma D, Coulibaly D, Diabaté A, Belemvire A, Diouf M, et al. Evidence supporting deployment of next generation insecticide treated nets in Burkina Faso: bioassays with either chlorfenapyr or piperonyl butoxide increase mortality of pyrethroid-resistant *Anopheles**gambiae**s.l*. Malaria J. 2021. 10.1186/s12936-021-03936-3. 10.1186/s12936-021-03936-3 PMC8524873 34663348

[9] Knols BGJ, Farenhorst M, Andriessen R, Snetselaar J, Suer RA, Osinga AJ, et al. Eave tubes for malaria control in Africa: an introduction. Malar J. 2016;15:404. 27515306 10.1186/s12936-016-1452-x PMC4982263

[10] Fiorenzano JM, Koehler PG, Xue R-D. Attractive toxic sugar bait (ATSB) for control of mosquitoes and its impact on non-target organisms: a review. Int J Environ Res Public Health. 2017;14:398. 28394284 10.3390/ijerph14040398 PMC5409599

[11] Ngufor C, Fongnikin A, Rowland M, N’Guessan R. Indoor residual spraying with a mixture of clothianidin (a neonicotinoid insecticide) and deltamethrin provides improved control and long residual activity against pyrethroid resistant *Anopheles gambiae* sl in Southern Benin. PLoS ONE. 2017;12:e0189575. 29252986 10.1371/journal.pone.0189575 PMC5734732

[12] Fongnikin A, Houeto N, Agbevo A, Odjo A, Syme T, N’Guessan R, et al. Efficacy of fludora® fusion (a mixture of deltamethrin and clothianidin) for indoor residual spraying against pyrethroid-resistant malaria vectors: laboratory and experimental hut evaluation. Parasit Vectors. 2020;13:466. 32917255 10.1186/s13071-020-04341-6 PMC7488472

[13] Ingham VA, Grigoraki L, Ranson H. Pyrethroid resistance mechanisms in the major malaria vector species complex. Entomologia Generalis. 2023;43:515–26.

[14] Ingham VA, Wagstaff S, Ranson H. Transcriptomic meta-signatures identified in *Anopheles gambiae* populations reveal previously undetected insecticide resistance mechanisms. Nat Commun. 2018;9:5282. 30538253 10.1038/s41467-018-07615-x PMC6290077

[15] Yunta C, Grisales N, Nász S, Hemmings K, Pignatelli P, Voice M, et al. Pyriproxyfen is metabolized by P450s associated with pyrethroid resistance in *An. **gambiae*. Insect Biochem Mol Biol. 2016;78:50–7. 27613592 10.1016/j.ibmb.2016.09.001 PMC6399515

[16] Yunta C, Hemmings K, Stevenson B, Koekemoer LL, Matambo T, Pignatelli P, et al. Cross-resistance profiles of malaria mosquito P450s associated with pyrethroid resistance against WHO insecticides. Pestic Biochem Physiol. 2019;161:61–7. 31685198 10.1016/j.pestbp.2019.06.007

[17] Ingham VA, Nagi S. Genomic profiling of insecticide resistance in malaria vectors: Insights into molecular mechanisms. Res Square. 2024;526:207.

[18] Njoroge H, Van’t Hof A, Oruni A, Pipini D, Nagi SC, Lynd A, et al. Identification of a rapidly-spreading triple mutant for high-level metabolic insecticide resistance in *Anopheles gambiae* provides a real-time molecular diagnostic for antimalarial intervention deployment. Mol Ecol. 2022;31:4307–18. 35775282 10.1111/mec.16591 PMC9424592

[19] Ibrahim SS, Riveron JM, Stott R, Irving H, Wondji CS. The cytochrome P450 CYP6P4 is responsible for the high pyrethroid resistance in knockdown resistance-free *Anopheles arabiensis*. Insect Biochem Mol Biol. 2015;68:23–32. 26548743 10.1016/j.ibmb.2015.10.015 PMC4717123

[20] Mugenzi LMJ, Menze BD, Tchouakui M, Wondji MJ, Irving H, Tchoupo M, et al. Cis-regulatory CYP6P9b P450 variants associated with loss of insecticide-treated bed net efficacy against *Anopheles **funestus*. Nature Commun. 2019. 10.1038/s41467-019-12686-5. 10.1038/s41467-019-12686-5 PMC6789023 31604938

[21] Aravindan V, Muthukumaravel S, Gunasekaran K. Interaction affinity of Delta and Epsilon class glutathione-s-transferases (GSTs) to bind with DDT for detoxification and conferring resistance in *Anopheles gambiae*, a malaria vector. J Vector Borne Dis. 2014;51:8–15. 24717196

[22] Pignatelli P, Ingham VA, Balabanidou V, Vontas J, Lycett G, Ranson H. The *Anopheles gambiae* ATP-binding cassette transporter family: phylogenetic analysis and tissue localization provide clues on function and role in insecticide resistance. Insect Mol Biol. 2018;27:110–22. 29068552 10.1111/imb.12351

[23] Logan RAE, Mäurer JB, Wapler C, Ingham VA. Uridine diphosphate (UDP)-glycosyltransferases (UGTs) are associated with insecticide resistance in the major malaria vectors *Anopheles**gambiae* s.l and *Anopheles**funestus*. Sci Rep. 2024;14:19821. 39191827 10.1038/s41598-024-70713-y PMC11350197

[24] Isaacs AT, Mawejje HD, Tomlinson S, Rigden DJ, Donnelly MJ. Genome-wide transcriptional analyses in *Anopheles* mosquitoes reveal an unexpected association between salivary gland gene expression and insecticide resistance. BMC Genomics. 2018;19:225. 29587635 10.1186/s12864-018-4605-1 PMC5870100

[25] Ingham VA, Anthousi A, Douris V, Harding NJ, Lycett G, Morris M, et al. A sensory appendage protein protects malaria vectors from pyrethroids. Nature. 2019;577:376–80. 31875852 10.1038/s41586-019-1864-1 PMC6974402

[26] Martinez-Torres D, Chandre F, Williamson MS, Darriet F, Bergé JB, Devonshire AL, et al. Molecular characterization of pyrethroid knockdown resistance (kdr) in the major malaria vector *Anopheles**gambiae**s.s*. Insect Mol Biol. 1998;7:179–84. 9535162 10.1046/j.1365-2583.1998.72062.x

[27] Clarkson CS, Miles A, Harding NJ, O’Reilly AO, Weetman D, Kwiatkowski D, et al. The genetic architecture of target-site resistance to pyrethroid insecticides in the African malaria vectors *Anopheles gambiae* and *Anopheles coluzzii*. Mol Ecol. 2021;30:5303–17. 33590926 10.1111/mec.15845 PMC9019111

[28] Weill M, Malcolm C, Chandre F, Mogensen K, Berthomieu A, Marquine M, et al. The unique mutation in *ace-1* giving high insecticide resistance is easily detectable in mosquito vectors. Insect Mol Biol. 2004;13:1–7. 14728661 10.1111/j.1365-2583.2004.00452.x

[29] Dada N, Lol JC, Benedict AC, López F, Sheth M, Dzuris N, et al. Pyrethroid exposure alters internal and cuticle surface bacterial communities in *Anopheles albimanus*. ISME J. 2019;13:2447–64. 31171859 10.1038/s41396-019-0445-5 PMC6776023

[30] Dada N, Sheth M, Liebman K, Pinto J, Lenhart A. Whole metagenome sequencing reveals links between mosquito microbiota and insecticide resistance in malaria vectors. Sci Rep. 2018. 10.1038/s41598-018-20367-4. 29391526 10.1038/s41598-018-20367-4 PMC5794770

[31] Omoke D, Kipsum M, Otieno S, Esalimba E, Sheth M, Lenhart A, et al. Western Kenyan *Anopheles **gambiae* showing intense permethrin resistance harbour distinct microbiota. Malaria J. 2021. 10.1186/s12936-021-03606-4. 10.1186/s12936-021-03606-4 PMC7869237 33557825

[32] Pelloquin B, Kristan M, Edi C, Meiwald A, Clark E, Jeffries CL, et al. Overabundance of *Asaia* and *Serratia* bacteria is associated with deltamethrin insecticide susceptibility in *Anopheles **coluzzii* from Agboville. Côte d’Ivoire Microbiol Spectr. 2021. 10.1128/Spectrum.00157-21. 34668745 10.1128/Spectrum.00157-21 PMC8528120

[33] Ingham VA, Tennessen JA, Lucas ER, Elg S, Yates HC, Carson J, et al. Integration of whole genome sequencing and transcriptomics reveals a complex picture of the reestablishment of insecticide resistance in the major malaria vector *Anopheles coluzzii*. PLoS Genet. 2021;17:e1009970. 34941884 10.1371/journal.pgen.1009970 PMC8741062

[34] Djondji Kamga FM, Mugenzi LMJ, Tchouakui M, Sandeu MM, Maffo CG, Nyegue MA, et al. Contrasting patterns of *Asaia* association with pyrethroid resistance escalation between the malaria vectors *Anopheles **funestus* and *Anopheles **gambiae*. Microorganisms. 2023;11:644. 36985217 10.3390/microorganisms11030644 PMC10053915

[35] Barnard K, Jeanrenaud ACSN, Brooke BD, Oliver SV. The contribution of gut bacteria to insecticide resistance and the life histories of the major malaria vector *Anopheles **arabiensis* (Diptera: Culicidae). Sci Rep. 2019. 10.1038/s41598-019-45499-z. 31235803 10.1038/s41598-019-45499-z PMC6591418

[36] President’s Malaria Initiative Ethiopia (PMI). Malaria Operational Plan. 2019.

[37] Williams J, Ingham VA, Morris M, Toé KH, Hien AS, Morgan JC, et al. Sympatric populations of the *Anopheles **gambiae* complex in southwest Burkina Faso evolve multiple diverse resistance mechanisms in response to intense selection pressure with pyrethroids. Insects. 2022;13:247. 35323544 10.3390/insects13030247 PMC8955173

[38] Williams J, Flood L, Praulins G, Ingham VA, Morgan J, Lees RS, et al. Characterisation of *Anopheles* strains used for laboratory screening of new vector control products. Parasit Vectors. 2019;12:522. 31690332 10.1186/s13071-019-3774-3 PMC6833243

[39] Witzig C, Parry M, Morgan J, Irving H, Steven A, Cuamba N, et al. Genetic mapping identifies a major locus spanning P450 clusters associated with pyrethroid resistance in kdr-free *Anopheles arabiensis* from Chad. Heredity. 2013;110:389–97. 23299100 10.1038/hdy.2012.112 PMC3607182

[40] WHO. Test procedures for insecticide resistance monitoring in malaria vector mosquitoes. Geneva: World Health Organization; 2016.

[41] Santolamazza F, Mancini E, Simard F, Qi Y, Tu Z, Della TA. Insertion polymorphisms of SINE200 retrotransposons within speciation islands of *Anopheles **gambiae* molecular forms. Malaria J. 2008. 10.1186/1475-2875-7-163. 10.1186/1475-2875-7-163 PMC2546427 18724871

[42] Bass C, Nikou D, Donnelly MJ, Williamson MS, Ranson H, Ball A, et al. Detection of knockdown resistance (*kdr*) mutations in *Anopheles gambiae*: a comparison of two new high-throughput assays with existing methods. Malar J. 2007;6:111. 17697325 10.1186/1475-2875-6-111 PMC1971715

[43] Bass C, Nikou D, Vontas J, Williamson MS, Field LM. Development of high-throughput real-time PCR assays for the identification of insensitive acetylcholinesterase (*ace-1*^R^) in *Anopheles gambiae*. Pestic Biochem Physiol. 2010;96:80–5.

[44] Livak KJ. Organization and mapping of a sequence on the *Drosophila melanogaster* X and Y chromosomes that is transcribed during spermatogenesis. Genetics. 1984;107:611–34. 6430749 10.1093/genetics/107.4.611 PMC1202380

[45] Dixon P. VEGAN, a package of R functions for community ecology. J Veg Sci. 2003;14:927–30.

[46] Kim D, Langmead B, Salzberg SL. HISAT: a fast spliced aligner with low memory requirements. Nat Methods. 2015;12:357–60. 25751142 10.1038/nmeth.3317 PMC4655817

[47] Liao Y, Smyth GK, Shi W. featureCounts: an efficient general purpose program for assigning sequence reads to genomic features. Bioinformatics. 2014;30:923–30. 24227677 10.1093/bioinformatics/btt656

[48] Love MI, Huber W, Anders S. Moderated estimation of fold change and dispersion for RNA-seq data with DESeq2. Genome Biol. 2014;15:550. 25516281 10.1186/s13059-014-0550-8 PMC4302049

[49] Giraldo-Calderón GI, Emrich SJ, MacCallum RM, Maslen G, Dialynas E, Topalis P, et al. VectorBase: an updated bioinformatics resource for invertebrate vectors and other organisms related with human diseases. Nucleic Acids Res. 2015;43:D707–13. 25510499 10.1093/nar/gku1117 PMC4383932

[50] Gimonneau G, Tchioffo MT, Abate L, Boissière A, Awono-Ambéné PH, Nsango SE, et al. Composition of *Anopheles coluzzii* and *Anopheles gambiae* microbiota from larval to adult stages. Infect Genet Evol. 2014;28:715–24. 25283802 10.1016/j.meegid.2014.09.029

[51] Vontas J, Grigoraki L, Morgan J, Tsakireli D, Fuseini G, Segura L, et al. Rapid selection of a pyrethroid metabolic enzyme CYP9K1 by operational malaria control activities. Proc Natl Acad Sci. 2018;115:4619–24. 29674455 10.1073/pnas.1719663115 PMC5939083

[52] Balabanidou V, Kampouraki A, MacLean M, Blomquist GJ, Tittiger C, Juárez MP, et al. Cytochrome P450 associated with insecticide resistance catalyzes cuticular hydrocarbon production in *Anopheles gambiae*. Proc Natl Acad Sci. 2016;113:9268–73. 27439866 10.1073/pnas.1608295113 PMC4995928

[53] Kefi M, Balabanidou V, Sarafoglou C, Charamis J, Lycett G, Ranson H, et al. ABCH2 transporter mediates deltamethrin uptake and toxicity in the malaria vector *Anopheles coluzzii*. PLoS Pathog. 2023;19:e1011226. 37585450 10.1371/journal.ppat.1011226 PMC10461823

[54] Messenger LA, Impoinvil LM, Derilus D, Yewhalaw D, Irish S, Lenhart A. A whole transcriptomic approach provides novel insights into the molecular basis of organophosphate and pyrethroid resistance in *Anopheles arabiensis* from Ethiopia. Insect Biochem Mol Biol. 2021;139:103655. 34562591 10.1016/j.ibmb.2021.103655 PMC11705372

[55] Müller P, Warr E, Stevenson BJ, Pignatelli PM, Morgan JC, Steven A, et al. Field-caught permethrin-resistant *Anopheles **gambiae* overexpress CYP6P3, a P450 that metabolises pyrethroids. PLoS Genet. 2008;4:e1000286. 19043575 10.1371/journal.pgen.1000286 PMC2583951

[56] Mitchell SN, Stevenson BJ, Müller P, Wilding CS, Egyir-Yawson A, Field SG, et al. Identification and validation of a gene causing cross-resistance between insecticide classes in *Anopheles **gambiae* from Ghana. Proc Natl Acad Sci. 2012;109:6147–52. 22460795 10.1073/pnas.1203452109 PMC3341073

[57] Williams J, Ingham VA, Morris M, Toé KH, Hien AS, Morgan JC, et al. Sympatric populations of the *Anopheles gambiae* complex in Southwest Burkina Faso evolve multiple diverse resistance mechanisms in response to intense selection pressure with pyrethroids. Insects. 2022;13:247. 35323544 10.3390/insects13030247 PMC8955173

[58] Mawejje HD, Weetman D, Epstein A, Lynd A, Opigo J, Maiteki-Sebuguzi C, et al. Characterizing pyrethroid resistance and mechanisms in *Anopheles**gambiae* (s.s) and *Anopheles**arabiensis* from 11 districts in Uganda. Current Res Parasitol Vector-Borne Dis. 2023;3:100106. 10.1016/j.crpvbd.2022.100106 PMC9798136 36590346

[59] Nash RK, Lambert Nguessan B, Ngufor R, Rowland C, Oxborough M, et al. Systematic review of the entomological impact of insecticide-treated nets evaluated using experimental hut trials in Africa. Current Res Parasitol Vector-Borne Dis. 2021;1:100047. 10.1016/j.crpvbd.2021.100047 PMC8906077 35284856

[60] Messenger LA, Shililu J, Irish SR, Anshebo GY, Tesfaye AG, Ye-Ebiyo Y, et al. Insecticide resistance in *Anopheles arabiensis* from Ethiopia (2012–2016): a nationwide study for insecticide resistance monitoring. Malar J. 2017;16:469. 29151024 10.1186/s12936-017-2115-2 PMC5694167

[61] Williams J, Cowlishaw R, Sanou A, Ranson H, Grigoraki L. In vivo functional validation of the V402L voltage gated sodium channel mutation in the malaria vector *An**gambiae*. Pest Manag Sci. 2022;78:1155–63. 34821465 10.1002/ps.6731

[62] Grigoraki L, Cowlishaw R, Nolan T, Donnelly M, Lycett G, Ranson H. CRISPR/Cas9 modified *An**gambiae* carrying kdr mutation L1014F functionally validate its contribution in insecticide resistance and combined effect with metabolic enzymes. PLOS Genet. 2021;17:e1009556. 34228718 10.1371/journal.pgen.1009556 PMC8284791

[63] Guégan M, Zouache K, Démichel C, Minard G, Van Tran V, Potier P, et al. The mosquito holobiont: fresh insight into mosquito-microbiota interactions. Microbiome. 2018;6:49. 29554951 10.1186/s40168-018-0435-2 PMC5859429

[64] Osei-Poku J, Mbogo CM, Palmer WJ, Jiggins FM. Deep sequencing reveals extensive variation in the gut microbiota of wild mosquitoes from Kenya. Mol Ecol. 2012;21:5138–50. 22988916 10.1111/j.1365-294X.2012.05759.x

[65] Brettell LE, Hoque AF, Joseph TS, Dhokiya V, Hornett EA, Hughes GL, et al. Mosquitoes reared in distinct insectaries within an institution in close spatial proximity possess significantly divergent microbiomes. bioRxiv. 2024;17:e0011306.

[66] Wang H, Liu H, Peng H, Wang Y, Zhang C, Guo X, et al. A symbiotic gut bacterium enhances *Aedes albopictus* resistance to insecticide. PLoS Negl Trop Dis. 2022;16:e0010208. 35245311 10.1371/journal.pntd.0010208 PMC8896681

[67] Ramya SL, Venkatesan T, Murthy KS, Jalali SK, Varghese A. Degradation of acephate by *Enterobacter asburiae*, *Bacillus cereus* and *Pantoea agglomerans* isolated from diamondback moth *Plutella xylostella* (L), a pest of cruciferous crops. J Environ Biol. 2016;37:611–8. 27498509

[68] Kulkarni AG, Kaliwal BB. Bioremediation of Methomyl by *Escherichia coli*. In: Bidoia ED, Montagnolli RN, editors. Toxicity and biodegradation testing. New York: Springer, New York; 2018.

[69] Dash DM, Osborne WJ. Rapid biodegradation and biofilm-mediated bioremoval of organophosphorus pesticides using an indigenous *Kosakonia oryzae* strain-VITPSCQ3 in a vertical-flow packed bed biofilm bioreactor. Ecotoxicol Environ Saf. 2020;192:110290. 32058164 10.1016/j.ecoenv.2020.110290

[70] Weiss BL, Maltz MA, Vigneron A, Wu Y, Walter KS, O’Neill MB, et al. Colonization of the tsetse fly midgut with commensal *Kosakonia cowanii* Zambiae inhibits trypanosome infection establishment. PLoS Pathog. 2019;15:e1007470. 30817773 10.1371/journal.ppat.1007470 PMC6394900

[71] Oliver SV, Brooke BD. The role of oxidative stress in the longevity and insecticide resistance phenotype of the major malaria vectors *Anopheles arabiensis* and *Anopheles funestus*. PLoS ONE. 2016;11:e0151049. 26964046 10.1371/journal.pone.0151049 PMC4786153

[72] Budachetri K, Kumar D, Crispell G, Beck C, Dasch G, Karim S. The tick endosymbiont *Candidatus* Midichloria mitochondrii and selenoproteins are essential for the growth of *Rickettsia parkeri* in the Gulf Coast tick vector. Microbiome. 2018;6:141. 30103809 10.1186/s40168-018-0524-2 PMC6090677

[73] Crispell G, Budachetri K, Karim S. *Rickettsia parkeri* colonization in *Amblyomma maculatum*: the role of superoxide dismutases. Parasit Vectors. 2016;9:291. 27206371 10.1186/s13071-016-1579-1 PMC4873992

