# Supplementary material for: Insecticide resistant Anopheles from Ethiopia but not Burkina Faso show a microbiota composition shift upon insecticide exposure
Source: Parasit Vectors. 2025 Jan 20;18:17. doi: 10.1186/s13071-024-06638-2 (PMC11748507; doi:10.1186/s13071-024-06638-2)
Supplement: Supplementary file 1 — Additional file 1. Supplementary Figures [file 13071_2024_6638_MOESM1_ESM.docx]

**
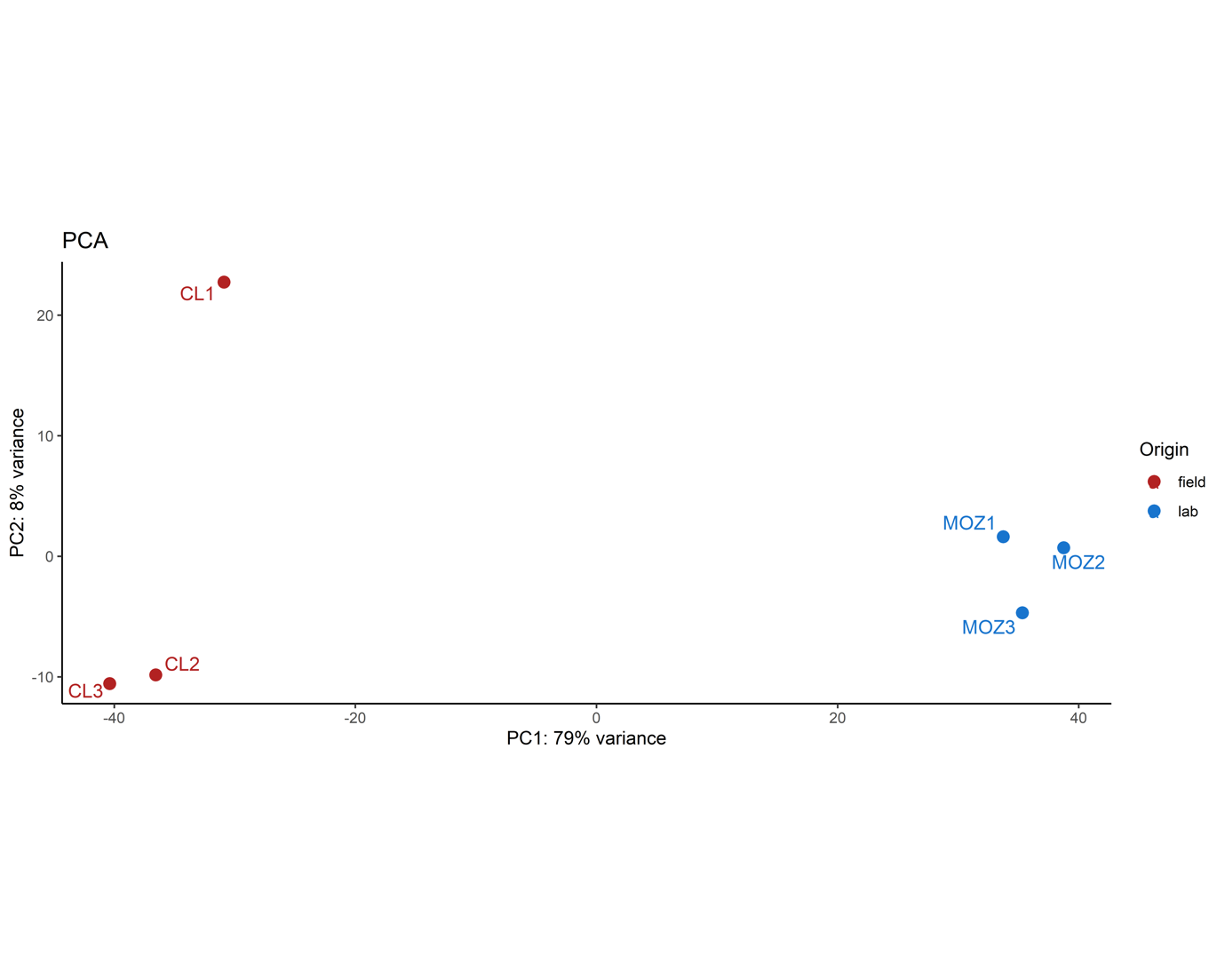
**

**Supplementary Figure 1: PCA of RNAseq samples.** PCA showing principal components (PC) one (x-axis) and two (y-axis) for read counts of Bahir Dar field samples (CL, red) and lab susceptible (Moz, blue). Variance for each PC is shown on the axis.

**
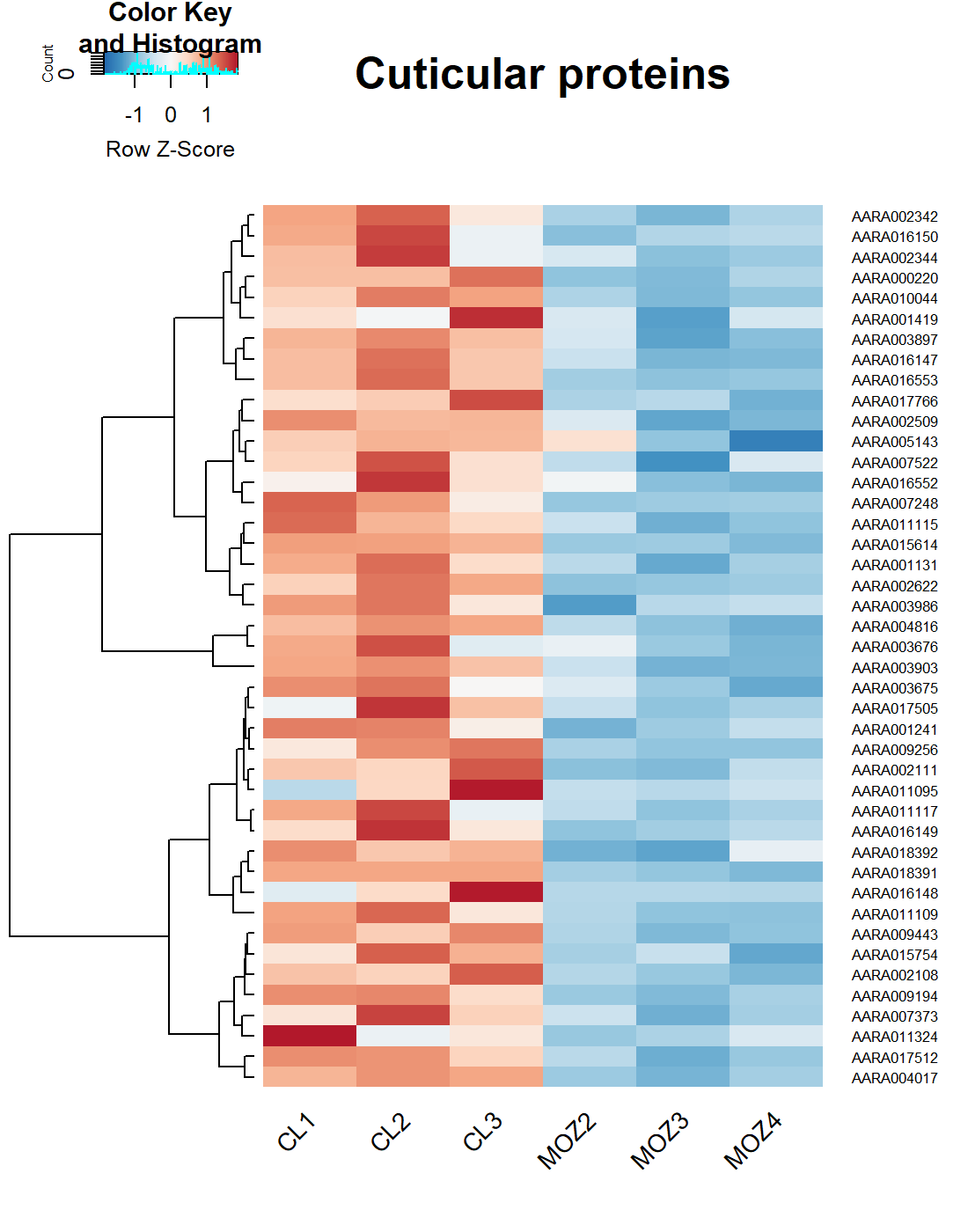
**

**Supplementary Figure 2: Significantly differential cuticular proteins.** Normalised read counts for Bahir Dar (CL) and lab susceptible (Moz) samples. Key is shown, with red demonstrating higher read counts and blue lower. The histogram on the key represents number of samples/genes within that Z-score. Histogram on the left shows distances of gene expression patterns.
